# The Law of Heritage in Regard to the Teeth and Jaws

**Published:** 1892-12

**Authors:** Henry S. Chase

**Affiliations:** St. Louis, Mo.


### THE LAW OF HERITAGE IN REGARD TO THE TEETH AND JAWS.[1]

BY HENRY S. CHASE, M.D., D.D.S., ST. LOUIS, MO.

THERE should be perfect harmony between the human teeth and the human jaws. Natural evolution of a race would at last produce this condition.

Naturalism is only the last term of a series, the first of which were inharmonious, and the succeeding ones less and less so, by the elimination of individual beings from the continuous line of descent. The free breeding of human beings on an island, or in an isolated country, would at last terminate in harmony between teeth and jaws, for adaptations to environment would continuously proceed until a level of agreement had been reached.

Fifty years ago I lived among a savage tribe of Indians in the then Northwest Territory, and now the State of Iowa. These people were called "The Sacs and Foxes." Their teeth were white and "even;" they were not crowded.

The "Winnebagoes," who inhabited a near-by portion of this country, were often seen on the "neutral grounds," and their teeth also were observed to be perfect.

---

[1] Read before the American Academy of Dental Science, Boston, June 1, 1892.

Naval expeditions for exploration of the islands of the world fifty and one hundred years ago, commanded by Cook and Wilkes, found the natives of the Pacific islands to possess perfect teeth as to beauty, soundness, and harmonious position in regard to each other and the jaws.

Why do the dentists of the United States find an entirely contrary condition in the patients whom they have to treat? It is because the American people are not a fixed breed. They are not yet Americanized.

Germans, Russians, Swedes, Polanders, Hungarians, Irish, Scotch, English, French, Italian, Spanish have divided their reproductive life-germs with each other, and produced children which will in some future time evolve a grand race of human beings. But this universal crossing of breeds without reference to the law of heritage is confusing enough to the scientist, but an utter despair to the mere student of the "practice of dentistry."

The dentist who has studied the science of heritage sees crowded but undecayed teeth in the mouth of a tall and beautiful girl of twenty years.

"Has your father sound teeth?"

"Yes."

"Has your mother sound teeth?"

"Yes."

"Are the teeth of your father regular and not crowded?"

"Yes."

"Are the teeth of your mother regular and not crowded?"

"Yes."

"How, then, is it that your own teeth are so dreadfully crowded?"

"I don't know. I wish you to straighten them."

The dentist continues: "Please let me ask you some questions. Where were your parents born?"

"My father was a gentleman's game-keeper in Germany, and my mother was the daughter of a shop-keeper in Paris."

"Is your father a tall, large man?"

"Yes."

"How is it with your mother?"

"Oh, she is very *petite;* she is only up to papa's shoulder."

Now, the man of science understands this. The girl has inherited the "locomotive system" of the father and the "vital system" of the mother. *Consequently she has inherited the teeth of her father and the jaws of her mother.* The father's teeth are large, and so are his jaws, and so there is harmony. The mother's teeth and jaws are small, and so in harmonious proportions. The law of heritage compels the jaws to go with the vital system. It also compels the teeth to go with the locomotive system. Thus large teeth are liable to grow in small jaws and small teeth in large jaws. The "locomotive system" gives the height of a person, and the "vital system" gives the rotundity of the person, including thus the organs of nutrition. This young lady is tall because she has her father's locomotive system, and she is thin because she has her mother's vital system. It is impossible for this father and mother to have a *petite* grown-up child.

Now, if this young lady has a sister not like herself, this sister will be just the opposite in conformation. For she will be short and rotund. She will have the height or "locomotive system" of her mother, and the rotundity or "vital system" of the father. Thus she ought to have small teeth in large jaws. On inquiry I found this to be true. "My sister has too much room for her teeth; her front teeth are too far apart." Further: "She is as short as my mother, but stouter." Further: "No, we do not look alike in form or feature, but we both look some like our parents, and it's funny." Now, you may see that if her mother had been short and stout, this young lady would have been of immense size,—"tall and big."

The dentists of America will, for a hundred or two years yet, have to deal with "irregularities," for hap-hazard marriages will still continue to take place, until the majority of people are educated in the law of resemblance and transmission of organizations. Nothing but "organization" is transmitted. There is no such thing as "blood" in breeds of beasts or humans. Either parent may give either the locomotive or the vital system. But one parent cannot give both systems. Each parent gives a defined half of the new being. Indeed, a definite half of himself or herself unite in the marriage of reproductive germ-cells to produce a child.

In the practical study of this subject we are confronted with "exceptions" to the law. If we had greater knowledge, discernment, perhaps we would understand that the "exceptions" are only seeming, and that an unseen factor is a part of the law of modification in "heritage." Perhaps "atavism" is the factor, recognizing by that word the persistent reproductive life-force from former generations.

The Indians of whom I have spoken were a pure breed of humans, *fixed* in their characteristics. Marriage out of their own tribe would only take one from the same family, and belonging to the same race. This is sufficient reason for harmony between their jaws and teeth.

This great family extended from the thirty-second to the forty-sixth degree, and from the Rocky Mountains east to the ocean.

